# MHD Casson carbon nanotube flow with mass and heat transfer under thermosolutal Marangoni convection in a porous medium: analytical solution

**DOI:** 10.1038/s41598-022-20532-w

**Published:** 2022-09-27

**Authors:** A. B. Vishalakshi, U. S. Mahabaleshwar, M. Hatami

**Affiliations:** 1 Department of Mathematics, Davangere University, Shivagangotri, Davangere, 577007 India; 2 Mechanical Engineering Department, Ferdowsi University of Mashhad, Mashhad, Iran

**Keywords:** Applied mathematics, Computational science, Other nanotechnology, Mechanical engineering

## Abstract

Current work portrays the flow of Marangoni convection Magneto hydrodynamics Casson fluid with carbon nanotubes under the effect of transpiration and radiation. The carbon nanotube particles namely water-single wall carbon nanotubes are inserted in the fluid to enhance better thermal efficiency. This type of flow problems is applicable for real life situations such as drying of silicon wafers, glues, crystal growth and heat exchangers and so on. The ordinary differential equations (ODEs) form of the result is yield to convert partial differential equations of the given equation by using similarity variables. Then this resulting ODEs are solved analytically, firstly using momentum equation to get solution domain and then by using this domain the energy equation solved to get the temperature profile in terms of Laguerre polynomial. Additionally, mass transpiration is also solved to get the concentration profile in terms of Laguerre polynomial. By using the different controlling parameters, the results can be discussed. And the effect of this parameters are discussed by using graphical arrangements. The newness of the present work is to explain the physically flow problem on the basis of chemically radiative thermosolutal Marangoni convective fluid.

## Introduction

Basically in the interfaces of liquid–liquid or liquid–gas we found the layers of Marangoni convection, these layers are normally called as dissipative layers and these layers plays a great role in industrial applications. Gibbs^1^ discovered this phenomenon in last century. Napolitano^2,3^ addressed the original work of this field. Temperature and concentration dependent surface tension is respectively called as Thermocapillary and destillocapillary effects^4,5^. The application of Marangoni convection can be found in the fields of crystal growth, soap films and crystal growth. Chamkha et al.^6^ worked on Marangoni convection problem and he come to concluded that surface driven flows may build layers along the interfaces as well as buoyancy effect brought on by gravity and the external pressure gradient. When employing the arc length as coordinates, Napolitano et al.^7^ addressed the issue that non-Marangoni boundary layers in bulk fluids do not explicitly depend on the geometry of the interface. Only a few research and initiatives have been made to comprehend the fundamental laws of nature and the issues surrounding Marangoni convection.

The magnetohydrodynamics thermosolutal Marangoni convection over a flat surface in the presence of a heat source/sink parameter was addressed by Mudhaf and Chamkha^8^. The effects of heat transmission on MHD and radiation are examined by Aly and Ebaid^9^. Marangoni boundary layer nanofluid led him to conclude that a magnetic parameter causes a fluid's velocity to slow down and its temperature to rise. The double-diffusive convection in an open cavity was studied by Arbin et al.^10^ Nayak^11^ investigated the magnetohydrodynamics viscoelastic fluid under the impact of chemical reaction effect with porous medium. See some other examples related to Marangoni convection are seen in^12–15^.

Recent advancements in nanotechnology helps to conduct innovative techniques to develop applications of nanofluids in many fields. The term nanofluid is initially addressed by Choi^16^. Arshad et al.^17–20^ investigated flow problems in the presence of different nanofluids with various aspects such as heat source/sink parameter, radiation and so on. See some more references on nanofluid in^21,22^. Similarly, carbon nanotubes take a lot of attention in many fields such as chemistry, physics, medicine, biology, and Engineering. These are the few examples for the importance of nanofluids in many fields^23–25^. Additionally, the fluid known as Casson fluid is quite interesting, and it is used to describe non-Newtonian phenomena. The researcher Casson addressed this flow model in 1995. This flow model is useful in many real life applications. See some of the recent works of this model in^26–28^.

Effect of Porous medium and thermal radiation take places major role in the fluid flow because these effects in the fluid flow is used in many industrial and real life applications namely metallurgic processes, geophysical and allied areas^29^. There are many equations and derivations are available to describe the fluid flow process through porous medium and also the effect of thermal radiation. See some more articles published on porous medium and thermal radiation are given in ^30–32^.

The current study is investigating Casson fluid flow in the presence of carbon nanotubes with thermal radiation and mass transpiration. It is prompted by the aforementioned studies. In this problem we use the new method to provide a similarity variable on the impact of chemically radiative thermosolutal Marangoni convective fluid flow, the partial differential equations of the governing equations are converted into ordinary differential equations. The novelty of the present work explains that the momentum energy and mass equation solved analytically to get the solution domain and the solution in terms of Laguerre polynomial. The impact of different parameters is examined with the help of graphical scenario. This work is also important in many industrial applications such as welding machines, metallurgical process, geosciences, space technology and so on. The current issue is persuasively argued in the work of Mahabaleshwar et al.^33^.

## Mathematical formulation and solution

**Figure 1:**
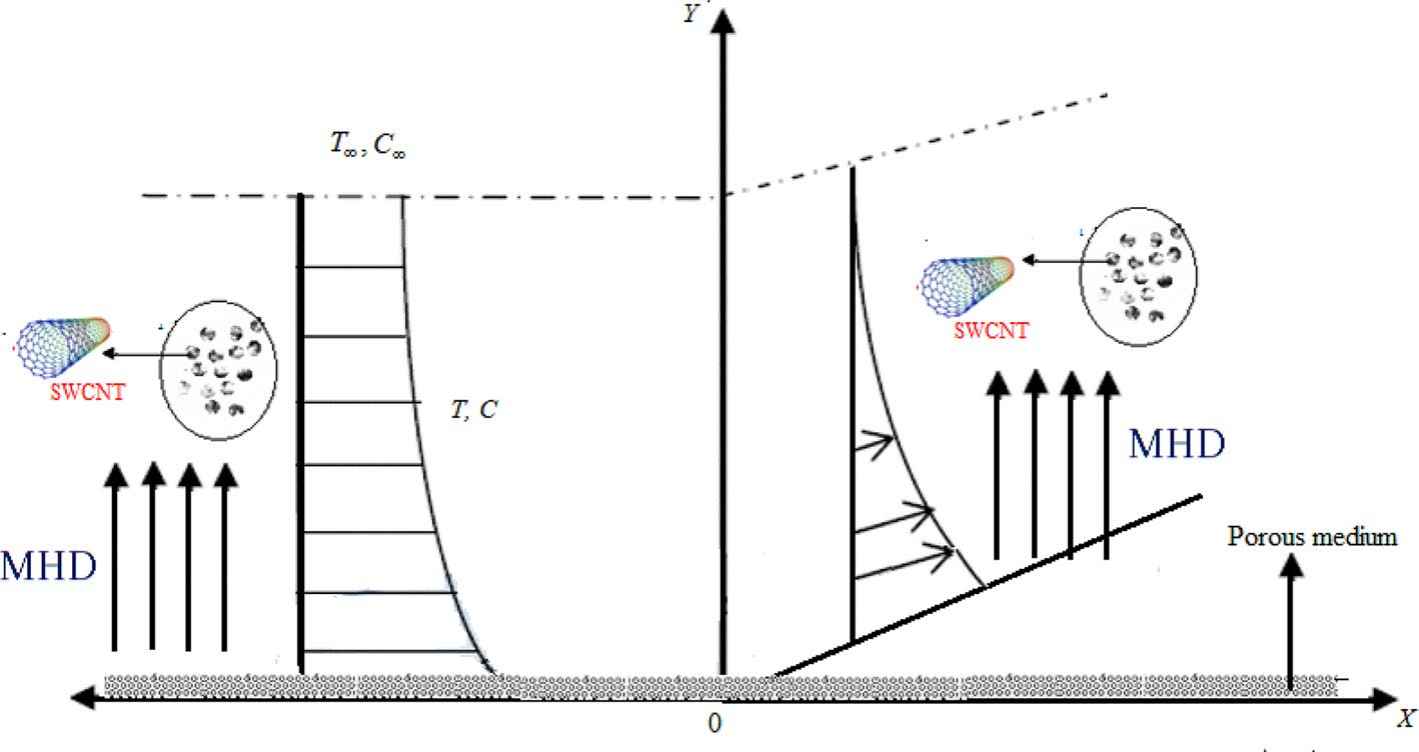
Schematic diagram of the Casson fluid flow.

Flow of a Casson fluid with thermosolutal Marangoni convection thermal radiation and transpiration is analyzed in this study. The particles of carbon nanotubes are immersed inside the fluid to get better thermal efficiency. Temperature gradients and solute concentrations define surface tension. Figure 1 shows a schematic representation of fluid flow.

Let us assume the surface of the fluid move towards *x* axis. The governing equations can be defined as follows by taking into account the aforementioned premises (See^34,35^).

1 $$\frac{\partial u}{{\partial x}} + \frac{\partial v}{{\partial y}} = 0,$$

2 $$u\frac{\partial u}{{\partial x}} + v\frac{\partial v}{{\partial y}} = \nu_{nf} \left( {1 + \frac{1}{\Lambda }} \right)\frac{{\partial^{2} u}}{{\partial y^{2} }} - \left( {\frac{{\mu_{nf} }}{{\rho_{nf} K}} + \frac{{\sigma_{nf} B_{0}^{2} }}{{\rho_{nf} }}} \right),$$

3 $$\begin{aligned} u\frac{\partial T}{{\partial x}} + v\frac{\partial T}{{\partial y}} = & \,\frac{{\kappa_{nf} }}{{\left( {\rho C_{P} } \right)_{nf} }}\frac{{\partial^{2} T}}{{\partial y^{2} }} + \frac{{\mu_{nf} }}{{\left( {\rho C_{P} } \right)_{nf} }}\left( {\frac{\partial u}{{\partial y}}} \right)^{2} + \left( {\frac{{\mu_{nf} }}{{\left( {\rho C_{P} } \right)_{nf} K}} + \frac{{\sigma_{nf} B_{0}^{2} }}{{\left( {\rho C_{P} } \right)_{nf} }}} \right)u^{2} \\ & - \,\frac{1}{{\left( {\rho C_{P} } \right)_{nf} }}\frac{{\partial q_{r} }}{\partial y} + \frac{{Q_{0} }}{{\left( {\rho C_{P} } \right)_{nf} }}\left( {T - T_{\infty } } \right), \\ \end{aligned}$$

4 $$u\frac{\partial C}{{\partial x}} + v\frac{\partial C}{{\partial y}} = D\frac{{\partial^{2} C}}{{\partial y^{2} }} - G\left( {C - C_{\infty } } \right),$$

Here the Casson fluid term is used for characterize the non-Newtonian fluid. Magnetic term and porous medium term is used for many scientific and technological phenomena. Effect of Porous medium and thermal radiation take places major role in the fluid flow because these effects in the fluid flow is used in many industrial and real life applications. Heat source/sink in the fluid flow influences the characteristics of heat transfer as there is a substantial amount of difference in the temperature between the surface and the fluid. Also the combination of mass transfer and heat source/sink helps in overcoming the problem of boundary layer separation.

The surface tension along with heat and mass boundaries is given by (See^36–38^)

5 $$\sigma = \sigma_{0} \left[ {1 - \gamma_{T} \left( {T - T_{\infty } } \right) - \gamma_{C} \left( {C - C_{\infty } } \right)} \right],$$

Coefficients of surface tension respectively for heat and mass is given by

6 $$\gamma_{T} = - \frac{1}{{\sigma_{0} }}\left( {\frac{\partial \sigma }{{\partial T}}} \right)_{T} ,\,\,\,\,{\text{and}}\,\,\,\gamma_{C} = - \frac{1}{{\sigma_{0} }}\left( {\frac{\partial \sigma }{{\partial C}}} \right)_{T} .$$

The terms from Eqs. (1–6) are specified in this section under Nomenclature.

Associated B. Cs

7 $$\mu \left( {\frac{\partial u}{{\partial y}}} \right)_{y = 0} = - \left( {\frac{\partial \sigma }{{\partial x}}} \right)_{y = 0} = \sigma_{0} \left( {\gamma_{T} \left( {\frac{\partial T}{{\partial x}}} \right)_{y = 0} + \gamma_{C} \left( {\frac{\partial C}{{\partial x}}} \right)_{y = 0} } \right),$$

8 $$\left. \begin{gathered} V\left( {x,0} \right) = V_{0} ,\,\,\,\,\,\,\,\,\,\,\,\,\,\,\,\,\,\,\,\,\,\,\,u\left( {x,\infty } \right) = 0 \hfill \\ T\left( {x,0} \right) = T_{\infty } + T_{0} X^{2} ,\,\,\,\,\,\,\,T\left( {x,\infty } \right) = T_{\infty } \hfill \\ C\left( {x,0} \right) = C_{\infty } + C_{0} X^{2} ,\,\,\,\,\,C\left( {x,\infty } \right) = C_{\infty } \hfill \\ \end{gathered} \right\}.$$

here $X = \frac{x}{L}$, and $L = - \frac{\mu \nu }{{\sigma_{0} T_{0} \gamma_{T} }}$ is the characteristic length, $T_{0} \,\,{\text{and}}\,\,C_{0}$ are constants. In addition, the following transformations are defined.

9 $$\left. {\begin{array}{*{20}l} {\psi \left( {x,y} \right) = \nu Xf\left( \eta \right)\,\,\,\,\eta = \frac{y}{L}} \hfill \\ {T\left( {x,y} \right) = T_{\infty } + T_{0} X^{2} \theta \left( \eta \right)} \hfill \\ {C = C_{\infty } + C_{0} X^{2} \phi \left( \eta \right)} \hfill \\ \end{array} } \right\},$$

By using dimensional form of velocity components are given by

10 $$u = \frac{\nu }{L}f_{\eta } \left( \eta \right),\,\,\,\,v = - \frac{\nu }{L}f\left( \eta \right).$$

The value $q_{r}$ can be defined on the basis of Rosseland’s approximation as follows (See^39–42^.

11 $$q_{r} = - \frac{{4\sigma^{*} }}{{3\alpha_{r} }}\frac{{\partial T^{4} }}{\partial y},$$

where the ambient temperature *T*^4^ expands in terms of the Taylor’s series as

12 $$T^{4} = T_{\infty }^{4} + 4T_{\infty }^{3} \left( {T - T_{\infty } } \right) + 6T_{\infty }^{2} \left( {T - T_{\infty } } \right)^{2} + \cdots$$

when the higher order elements in this equation are ignored, this results in

13 $$T^{4} \cong 3T_{\infty }^{3} + 4T_{\infty }^{3} T.$$

On applying Eq. (8) into Eq. (6), then the first order derivative of heat flux can be given by

14 $$\frac{{\partial q_{r} }}{\partial y} = - \frac{{16\sigma^{*} T_{\infty }^{3} }}{{3\alpha_{r} }}\frac{{\partial^{2} T}}{{\partial y^{2} }}.$$

Therefore, the Eq. (3) can b rewritten as

15 $$\begin{aligned} u\frac{\partial T}{{\partial x}} + v\frac{\partial T}{{\partial y}} = & \,\frac{{\kappa_{nf} }}{{\left( {\rho C_{P} } \right)_{nf} }}\frac{{\partial^{2} T}}{{\partial y^{2} }} + \frac{{\mu_{nf} }}{{\left( {\rho C_{P} } \right)_{nf} }}\left( {\frac{\partial u}{{\partial y}}} \right)^{2} + \left( {\frac{{\mu_{nf} }}{{\left( {\rho C_{P} } \right)_{nf} K}} + \frac{{\sigma_{nf} B_{0}^{2} }}{{\left( {\rho C_{P} } \right)_{nf} }}} \right)u^{2} \\ & \, + \frac{1}{{\left( {\rho C_{P} } \right)_{nf} }}\frac{{16\sigma^{*} T_{\infty }^{3} }}{{3k^{*} }}\frac{{\partial^{2} T}}{{\partial y^{2} }} + \frac{{Q_{0} }}{{\left( {\rho C_{P} } \right)_{nf} }}\left( {T - T_{\infty } } \right). \\ \end{aligned}$$

By using Eqs. (9) and (10) in Eqs. (2) and (3) to get

16 $$\varepsilon_{1} \left( {1 + \frac{1}{\Lambda }} \right)f_{\eta \eta \eta } + \varepsilon_{2} \left( {ff_{\eta \eta } - f_{\eta }^{2} } \right) - \left( {\varepsilon_{1} Da^{ - 1} + \varepsilon_{3} Q} \right)f_{\eta } = 0,$$

17 $$\left( {\varepsilon_{5} + R} \right)\theta_{\eta \eta } + \Pr \varepsilon_{4} \left( {f\theta_{\eta } + \left( {I - 2f_{\eta } } \right)\theta } \right) + Ec\left( {\varepsilon_{1} f_{\eta \eta }^{2} + \left( {\varepsilon_{1} Da^{ - 1} + \varepsilon_{3} Q} \right)f_{\eta } } \right) = 0,$$

18 $$\phi_{\eta \eta } + Sc\left( {f\phi_{\eta } - \left( {\delta + 2f_{\eta } } \right)\phi } \right) = 0,$$

the B. Cs reduces to

19 $$\begin{aligned} f\left( 0 \right) = & V_{C} ,\,\,\,f_{\eta } \left( \infty \right) = 0,\,\,\,\,\,\,\,\,f_{\eta \eta } \left( 0 \right) = - 2\left( {1 + M_{a} } \right) \\ \theta \left( 0 \right) = & 1,\,\,\,\theta \left( \infty \right) = 0,\,\,\,\phi \left( 0 \right) = 1,\,\,\,\phi \left( \infty \right) = 0, \\ \end{aligned}$$

here $V_{C} = - \frac{\gamma }{L}{\text{v}}_{0}$ is the mass transpiration, here $V_{C} = 0$, $V_{C} > 0$ and $V_{C} < 0$ respectively indicates suction, injection and no-permeability cases. $\Pr = \frac{\kappa }{{\mu C_{P} }},\,\,Sc = \frac{\nu }{D}\,\,{\text{and}}\,\delta = \frac{{GL^{2} }}{\nu }$ respectively indicates the Prandtl number, Schmidt number, chemical reaction coefficient. $R = \frac{{16\sigma^{*} T_{\infty }^{3} }}{{3\alpha_{r} \kappa }}$ is the radiation number, $I = \frac{{Q_{0} L^{2} }}{{\varepsilon_{4} \rho C_{P} \nu }}$ is the heat source or sink parameter, $Da^{ - 1} = \frac{{L^{2} }}{K}$ is inverse Darcy number, $Q = \frac{{\sigma B_{0}^{2} L^{2} }}{\rho \nu }$ is Chandrasekhar’s number, $Ec = \frac{{\gamma^{2} }}{{L^{2} T_{0} C_{P} }}$ is Eckert number, and finally $Ma = \frac{{Ma_{C} }}{{Ma_{T} }}$ is the Marangoni number (Thermosolutal surface tension ratio), $Ma_{C} = \frac{{\sigma_{0} \gamma_{C} C_{0} LC_{P} }}{\kappa }$ and $Ma_{T} = \frac{{\sigma_{0} \gamma_{T} T_{0} LC_{P} }}{\kappa }$ are the solutal and thermal Marangoni numbers. Carbon nanofluid quantities used in Eqs. (16) and (17) can be defined as (See^43,44^)

$$\varepsilon_{1} = \frac{{\mu_{nf} }}{{\mu_{f} }},\,\,\varepsilon_{2} = \frac{{\rho_{nf} }}{{\rho_{f} }},\,\,\varepsilon_{3} = \frac{{\sigma_{nf} }}{{\sigma_{f} }},\,\,\varepsilon_{4} = \frac{{\left( {\rho C_{P} } \right)_{nf} }}{{\left( {\rho C_{P} } \right)_{f} }},\,\,\varepsilon_{4} = \frac{{\kappa_{nf} }}{{\kappa_{f} }}$$

## Exact solutions

### Exact solution for momentum equation

Consider Eq. (16)’s solution, which has the following structure (See^45^)

20 $$f\left( \eta \right) = f_{\infty } + \left( {V_{C} - f_{\infty } } \right)Exp\left( { - \beta \eta } \right),$$

21 $${\text{here}},\,\,f_{\infty } = \beta - \frac{{Da^{ - 1} }}{\beta },$$

Although, from the governing B. Cs $f\left( 0 \right) = V_{C} ,\,\,\,f_{\eta } \left( \infty \right) = 0,\,\,{\text{and}}\,\,f_{\eta \eta } \left( 0 \right) = - 2\left( {1 + M_{a} } \right)$ is simultaneously satisfied $f_{\infty }$ for $\beta > 0$ is as follows

22 $$f_{\infty } = V_{C} + \frac{{2\left( {1 + M_{a} } \right)}}{{\beta^{2} }}.$$

On applying Eqs. (20, 21) in Eq. (16) to yield the following cubic equation

23 $$\varepsilon_{1} \left( {1 + \frac{1}{\Lambda }} \right)\beta^{3} - \varepsilon_{2} V_{C} \beta^{2} - \left( {\varepsilon_{1} Da^{ - 1} + \varepsilon_{3} Q} \right)a - 2\varepsilon_{2} \left( {1 + M_{a} } \right) = 0.$$

Then the velocity can be required as

24 $$f_{\eta } \left( \eta \right) = - \beta \left( {V_{C} - f_{\infty } } \right)Exp\left( { - \beta \eta } \right).$$

### Exact solution for temperature and concentration equation

For the purpose of solving temperature and concentration equation we introduce the following new variable for temperature and concertation respectively as follows

25 $$\xi = \left( {\frac{{\Pr \left( {V_{C} - f_{\infty } } \right)}}{{\beta \left( {\varepsilon_{5} + R} \right)}}} \right)Exp\left( { - \beta \eta } \right),\,\,{\text{for}}\,\,{\text{temperature}}$$

26 $$\varsigma = \left( {\frac{{Sc\left( {V_{C} - f_{\infty } } \right)}}{\beta }} \right)Exp\left( { - \beta \eta } \right),\,\,{\text{for}}\,\,{\text{concentration}}$$

Using Eqs. (25) and (26) respectively in Eqs. (17) and (18) to get

27 $$\xi \frac{{\partial^{2} \theta }}{{\partial \xi^{2} }} + \left( {1 - \varepsilon_{4} S_{1} - \varepsilon_{4} \xi } \right)\frac{\partial \theta }{{\partial \xi }} + \varepsilon_{4} \left( {2 - \frac{{\gamma_{1} }}{\xi }} \right)\theta = - Ec_{1} \xi ,$$

28 $$\varsigma \frac{{\partial^{2} \phi }}{{\partial \varsigma^{2} }} + \left( {1 - p - \varsigma } \right)\frac{\partial \phi }{{\partial \varsigma }} + \left( {2 + \frac{q}{\varsigma }} \right)\phi = 0,$$

here

$$\begin{aligned} & S_{1} = \frac{{\Pr f_{\infty } }}{{\beta \left( {\varepsilon_{5} + R} \right)}},\,\,\,\,\gamma_{1} = \frac{I\Pr }{{\beta \left( {\varepsilon_{5} + R} \right)}} \\ & Ec_{1} = - \frac{{Ec\beta^{2} }}{{\Pr^{2} }}\left( {\varepsilon_{5} + R} \right)\left( {\varepsilon_{1} \beta^{2} + \left( {\varepsilon_{1} Da^{ - 1} + \varepsilon_{3} Q} \right)} \right) \\ & p = \frac{{Scf_{\infty } }}{\beta }\,\,\,q = - \frac{Sc\delta }{{\beta^{2} }}, \\ \end{aligned}$$

The B. Cs are also reducing to

29 $$\theta \left( {\xi = - 1} \right) = 1,\,\,\,\,\theta \left( {\xi = 0} \right) = 0,$$

30 $$\phi \left( {\varsigma = - 1} \right) = 1,\,\,\,\,\phi \left( {\varsigma = 0} \right) = 0,$$

on solving Eqs. (27) and (28) by using Frobenius method to yield the following equations

31 $$\theta \left( \eta \right) = \left( {1 - A_{3} } \right)Exp\left( { - \beta A_{2} \eta } \right)\frac{{L\left( {2 - A_{2} ,\,\,A_{1} ,\,\,\varepsilon_{4} \xi } \right)}}{{L\left( {2 - A_{2} ,\,\,A_{1} ,\,\, - \varepsilon_{4} \xi_{0} } \right)}} + A_{3} \xi^{2} ,$$

32 $$\phi \left( \eta \right) = Exp\left( { - \beta B_{2} \eta } \right)\frac{{L\left( {2 - B_{2} ,\,\,B_{1} ,\,\,\varsigma } \right)}}{{L\left( {2 - B_{2} ,\,\,B_{1} ,\,\,\varsigma_{0} } \right)}}.$$

where

$$\begin{aligned} A_{1} = & \sqrt {\varepsilon_{4}^{2} S_{1}^{2} + 4\varepsilon_{4} \gamma_{1} } ,\,\,\,\,\,A_{2} = \frac{{\varepsilon_{4} S_{1} }}{2} + \frac{{\sqrt {\varepsilon_{4}^{2} S_{1}^{2} + 4\varepsilon_{4} \gamma_{1} } }}{2},\,\,\,\,A_{3} = \frac{{\varepsilon_{4} Ec_{1} }}{{\varepsilon_{4} \left( {2S_{1} + \gamma_{1} } \right) - 4}} \\ B_{1} = & \sqrt {P^{2} - 4q} ,\,\,\,\,\,B_{2} = \frac{P}{2} + \frac{{\sqrt {P^{2} - 4q} }}{2} \\ \xi_{0} = & \left( {\frac{{\Pr \left( {V_{C} - f_{\infty } } \right)}}{{\beta \left( {\varepsilon_{5} + R} \right)}}} \right),\,\,\,\,\,\,\,\,\varsigma_{0} = \left( {\frac{{Sc\left( {V_{C} - f_{\infty } } \right)}}{\beta }} \right) \\ \end{aligned}$$

## Results and discussion

This article portrays the Casson fluid flow with Marangoni convection with Carbon nanoparticles are immersed in the fluid flow to enhance the thermal efficiency of the fluid. Analytical results are examined with the help of different controlling parameters namely Casson fluid parameter, inverse Darcy number, Chandrasekhar’s number, Marangoni number and so on. The significant effect of Prandtl number, Schmidt number, chemically reaction coefficient and heat source/sink parameter on temperature, concentration and heat source/sink parameter is discussed as follows. The graphical scenario can be disused as follows.

**Figure 2:**
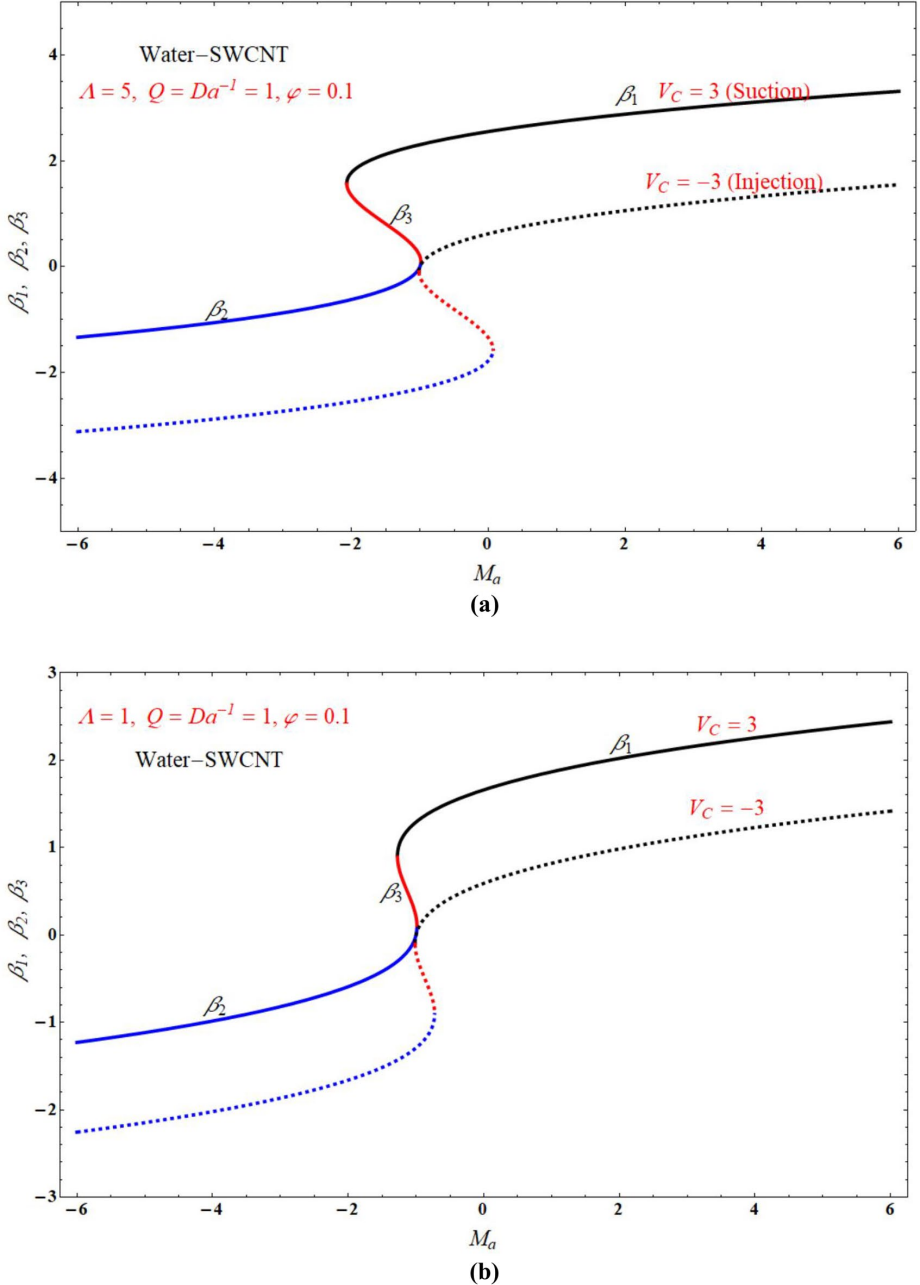
The plots of $\beta_{1} ,\,\beta_{2} ,\,\beta_{3}$ verses $Ma$ for suction and injection cases at (**a**) $\Lambda = 5$, and (**b**) $\Lambda = 1$.

**Figure 3:**
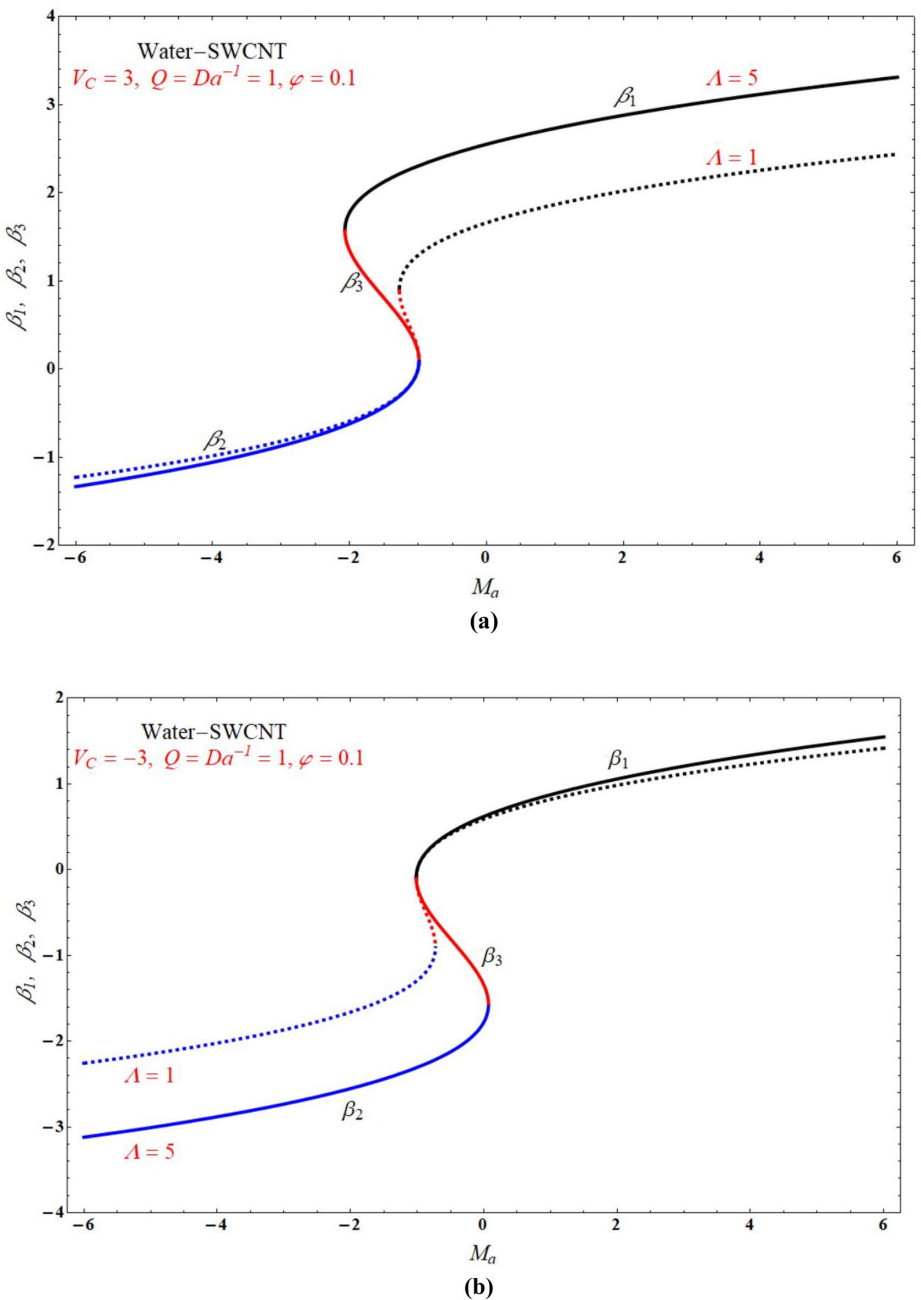
The plots of $\beta_{1} ,\,\beta_{2} ,\,\beta_{3}$ verses $Ma$ for $\Lambda = 5$ and $\Lambda = 1$ cases at (**a**) $V_{C} = 3$, and (**b**) $V_{C} = - 3$.

**Figure 4:**
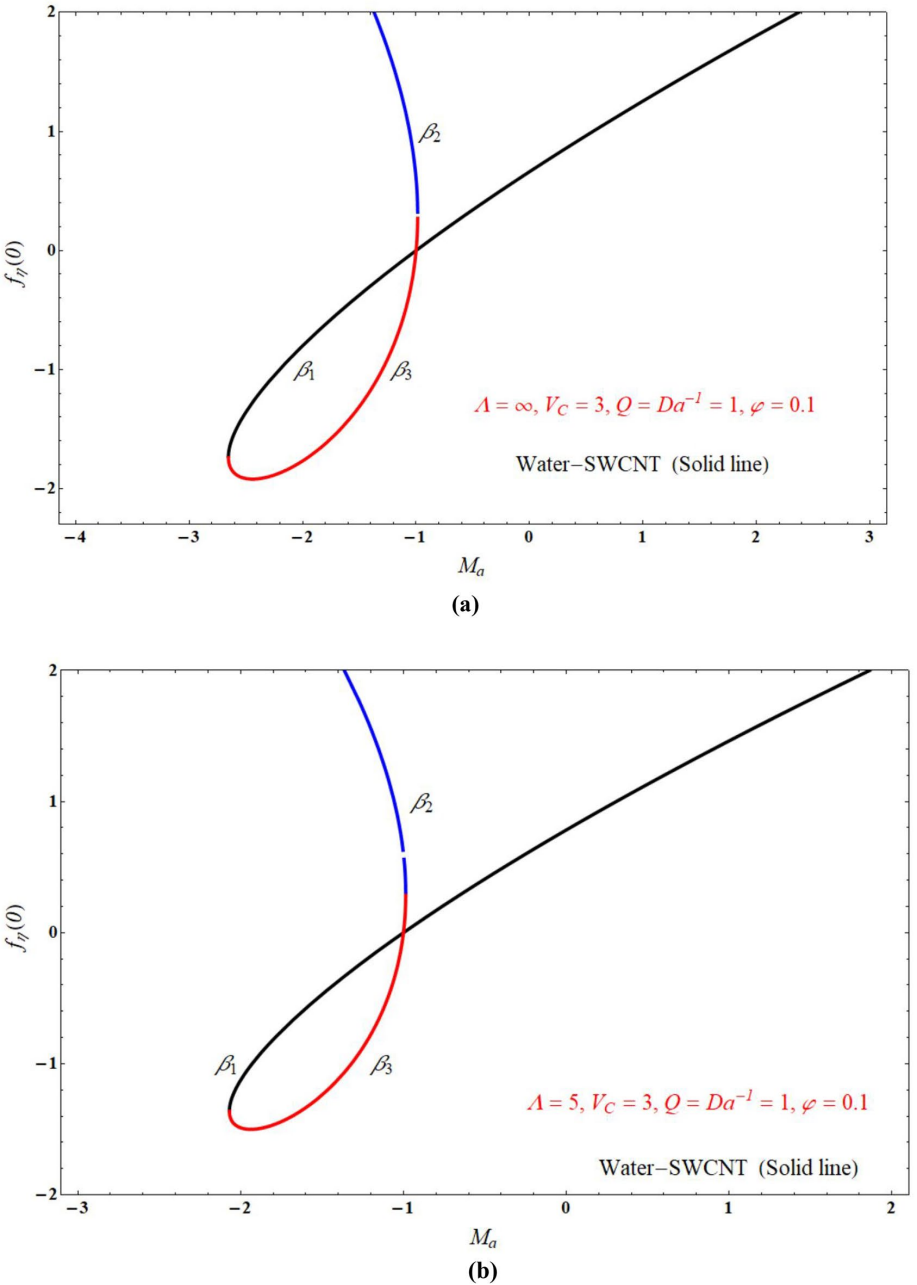
The plots of $f_{\eta } \left( 0 \right)$ verses $Ma$ at (a) $\Lambda = \infty$, and (b) $\Lambda = 5$.

**Figure 5:**
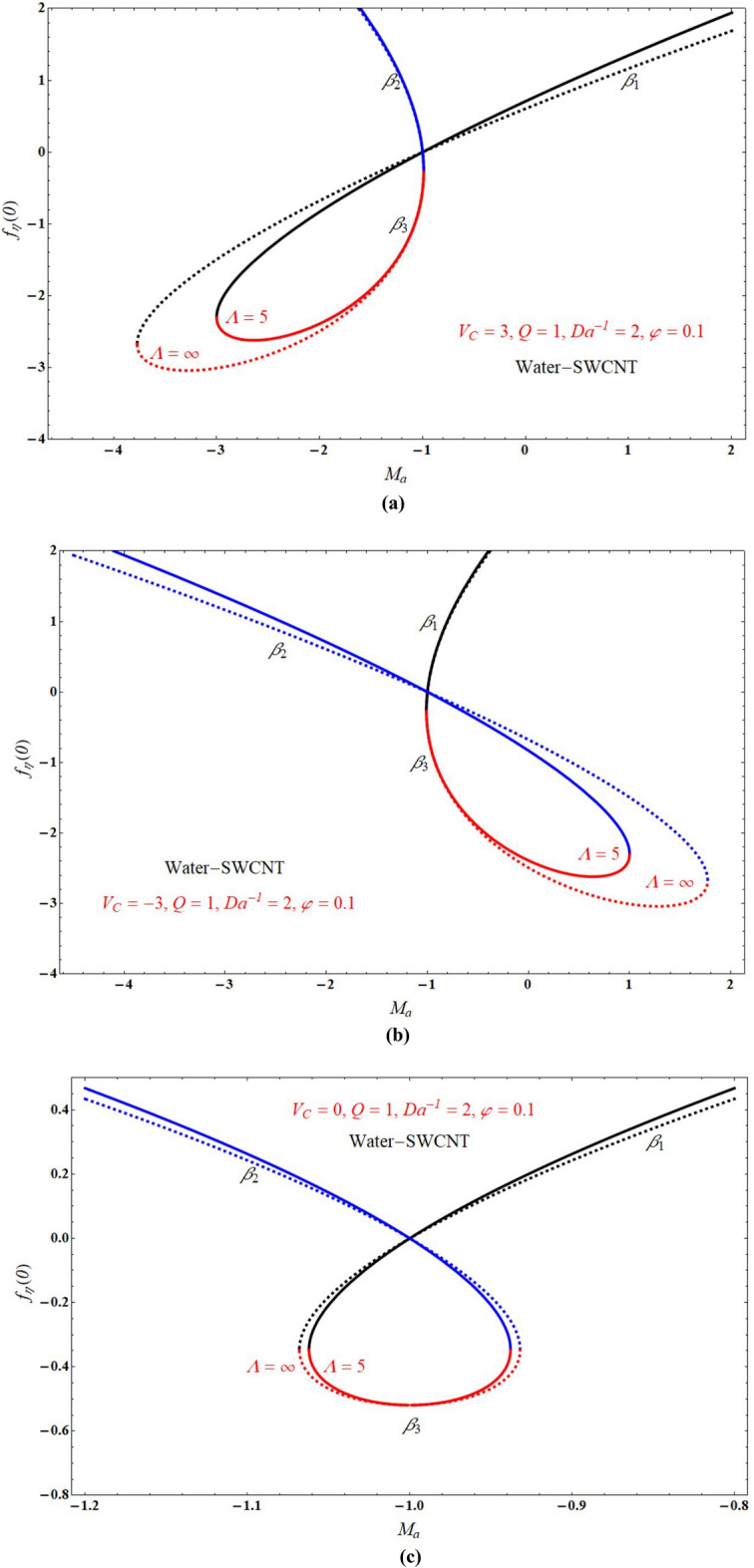
The plots of $f_{\eta } \left( 0 \right)$ verses $Ma$ for $\Lambda = 5$ and $\Lambda = \infty$ at (**a**) $V_{C} = 3$, (**b**) $V_{C} = - 3$ and (**c**) $V_{C} = 0$.

**Figure 6:**
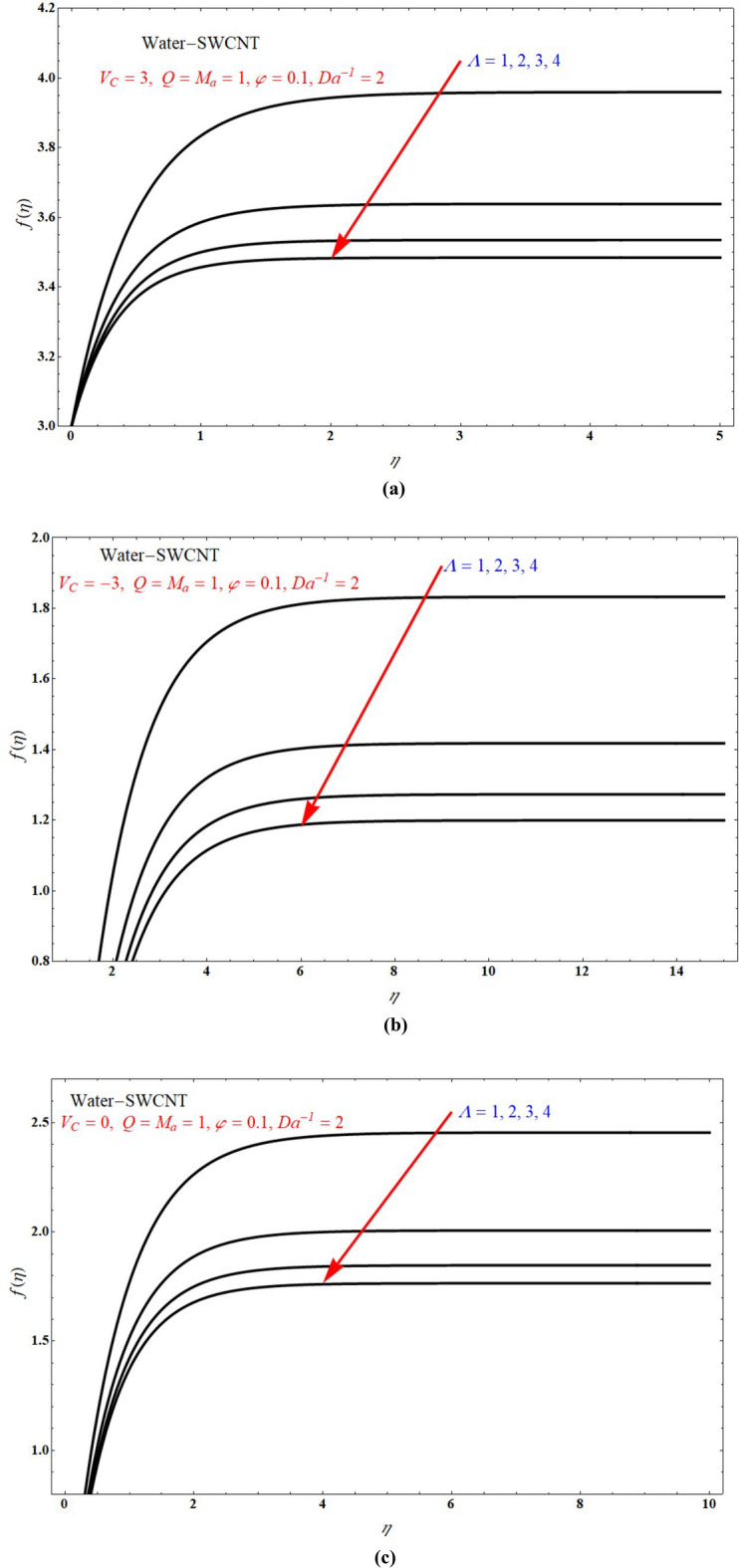
$f\left( \eta \right)$ verses $\eta$ for various values of $\Lambda$ at (**a**) $V_{C} = 3$, (**b**) $V_{C} = - 3$ and (**c**) $V_{C} = 0$.

**Figure 7:**
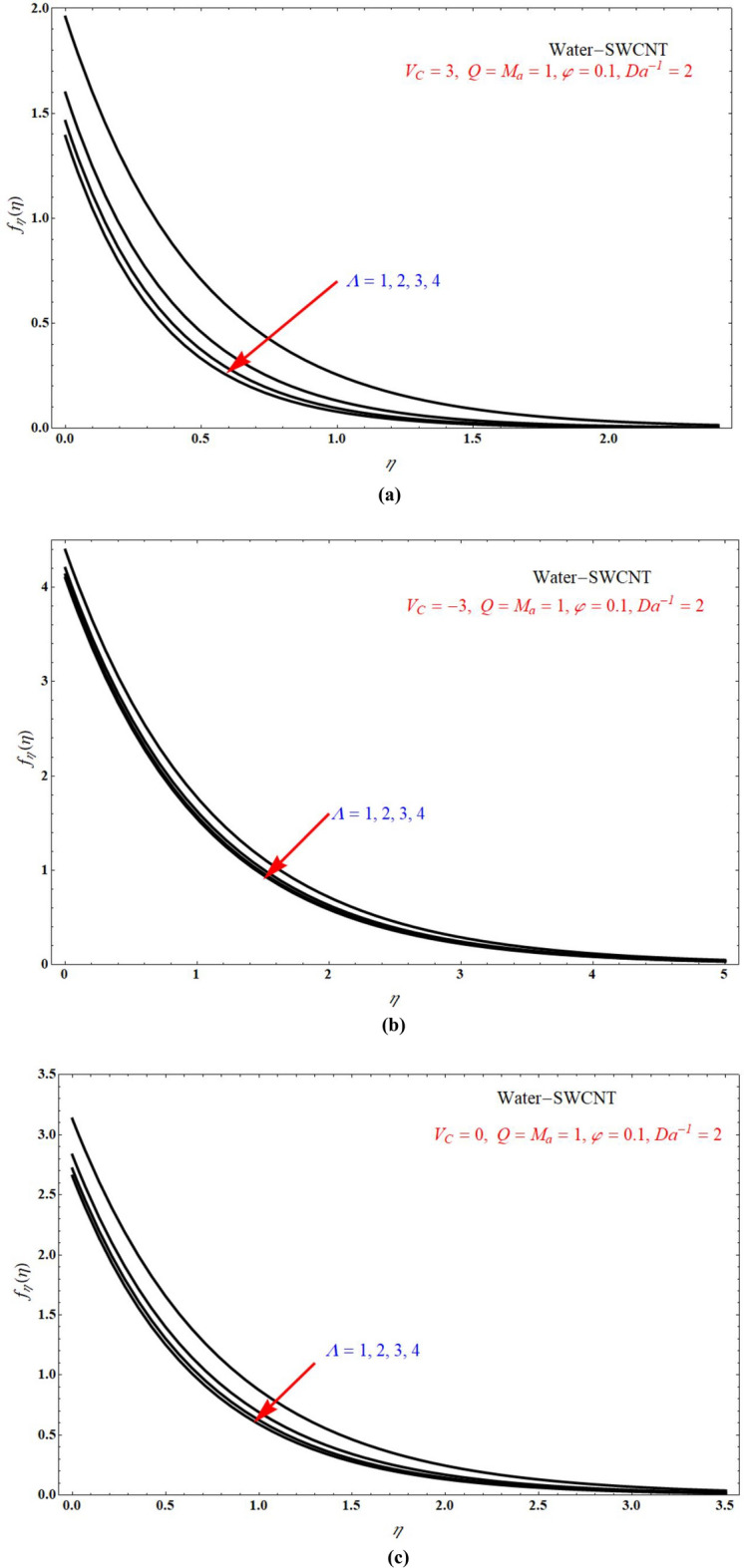
$f_{\eta } \left( \eta \right)$ verses $\eta$ for various values of $\Lambda$ at (**a**) $V_{C} = 3$, (**b**) $V_{C} = - 3$ and (**c**) $V_{C} = 0$.

Figure 2a,b demonstrated that the one of the results of Eq. (23), The red solid and dashed lines of the figure represents non-physical solution for various values of $Ma$ and keeping other parameters with suitable values. The effect of the physical solution varied directly with $V_{C}$, $Da^{ - 1}$ and $Ma$. Similarly, Fig. 3a,b portrays the plots of physical solution verses $Ma$ for different Casson fluid parameter $\Lambda$ for $V_{C} > 0$ and $V_{C} < 0$ cases respectively. Figures 4 and 5 represents the relation associated with velocity $f_{\eta } \left( 0 \right)$ with roots $\beta_{1} ,\,\beta_{2} \,\beta_{3}$ for various values of $Ma$. The physical and nonphysical surfaces depending upon the positive and negative roots respectively. From Fig. 4a,b we observe that the $\Lambda$ directly affected the surface velocity and $Ma$. Also from Fig. 5a–c it is cleared that the $V_{C}$ is directly affected the surface velocity and $Ma$. Figure 6 indicates $f\left( \eta \right)$ verses $\eta$ for different values of $\Lambda$. In this Fig. 6a represents suction case, Fig. 6b indicates injection case and Fig. 6c indicates no permeability case. Here it is cleared that $f\left( \eta \right)$ is less for more values of $\Lambda$ for both suction, injection and no permeability cases. Figure 7a–c indicates $f_{\eta } \left( \eta \right)$ verses $\eta$ for various values of $\Lambda$ for $V_{C} > 0$, $V_{C} < 0$ and $V_{C} = 0$ respectively. From this it is cleared that $f_{\eta } \left( \eta \right)$ decreases with increasing the values of $\Lambda$ for $V_{C} > 0$, $V_{C} < 0$ and $V_{C} = 0$.

**Figure 8:**
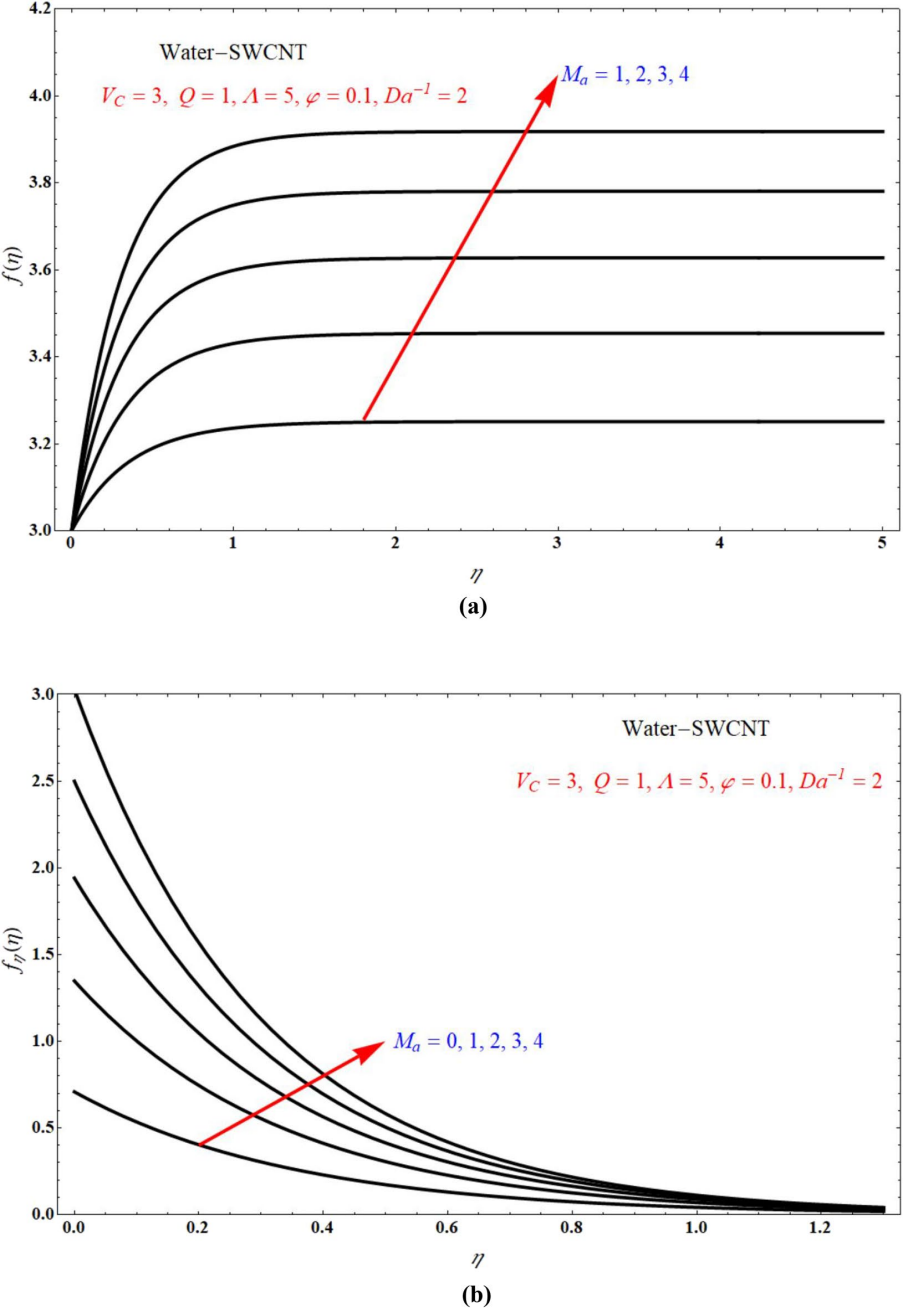
Plots of (**a**) $f\left( \eta \right)$ verses $\eta$ and (**b**) $f_{\eta } \left( \eta \right)$ verses $\eta$ for different choices of $M_{a}$ at suction case.

**Figure 9:**
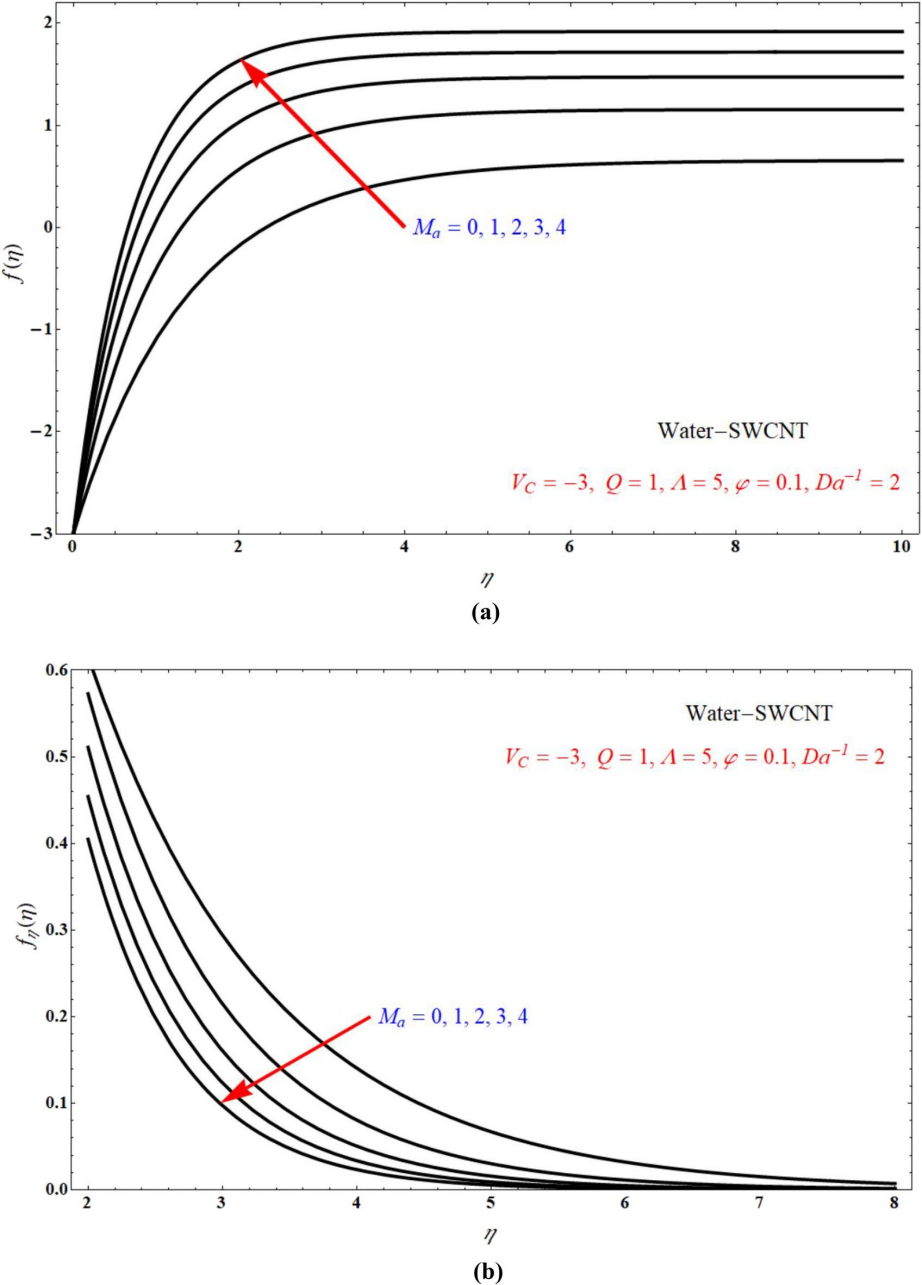
The plots of (**a**) $f\left( \eta \right)$ verses $\eta$ and (**b**) $f_{\eta } \left( \eta \right)$ verses $\eta$ for different choices of $M_{a}$ at injection case.

**Figure 10:**
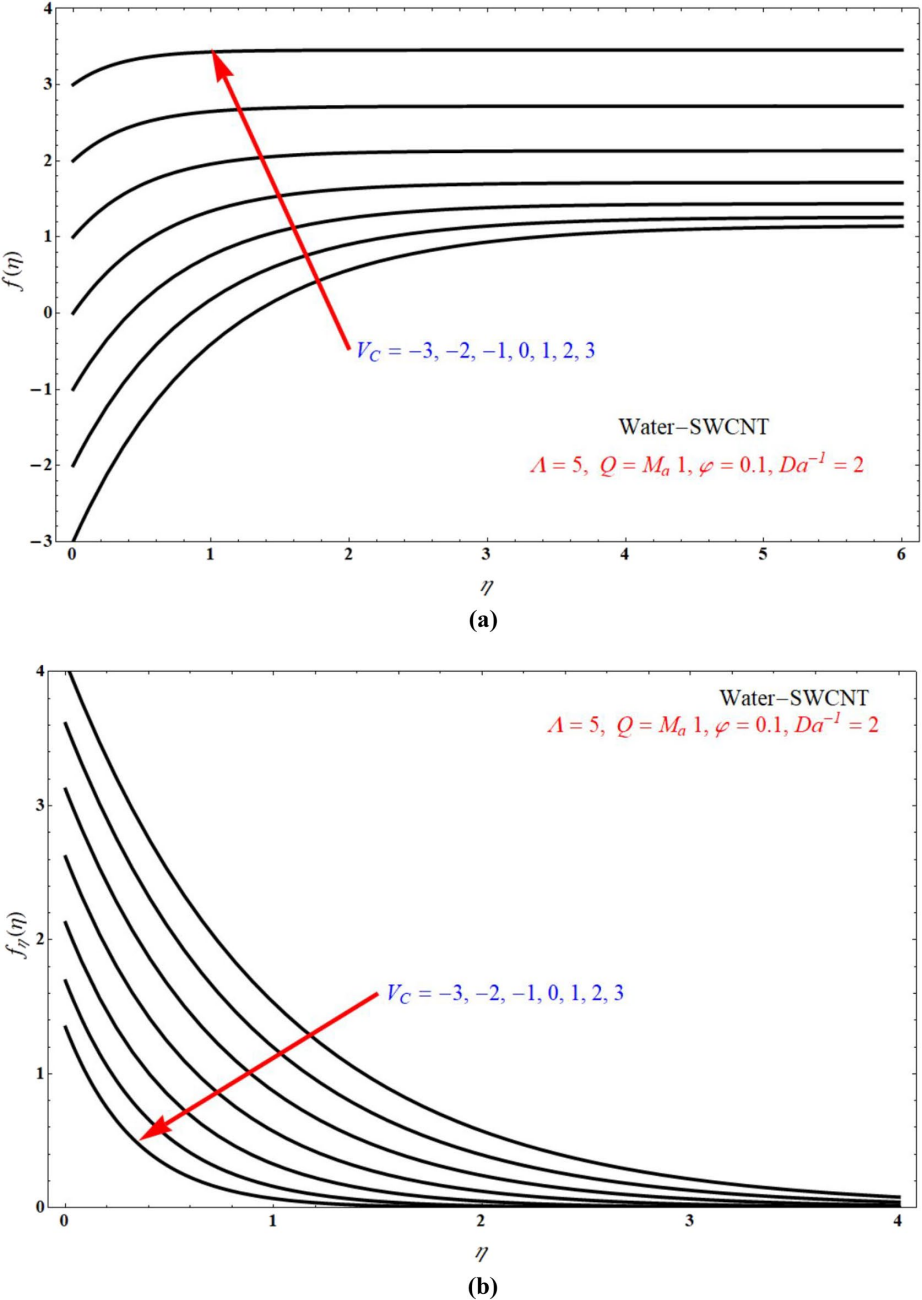
The plots of (**a**) $f\left( \eta \right)$ verses $\eta$ and (**b**) $f_{\eta } \left( \eta \right)$ verses $\eta$ for different choices of $V_{C}$.

Impact of $f\left( \eta \right)$ verses $\eta$ and $f_{\eta } \left( \eta \right)$ verses $\eta$ for various values of $M_{a}$ is respectively indicated at Fig. 8a,b for $V_{C} > 0$, and keeping all other parameters with suitable values. Here $f\left( \eta \right)$ and $f_{\eta } \left( \eta \right)$ is more for more values of $M_{a}$ for $V_{C} > 0$. Figure 9a,b indicates $f\left( \eta \right)$ verses $\eta$ and $f_{\eta } \left( \eta \right)$ verses $\eta$ for different values of $M_{a}$ at $V_{C} < 0$ respectively. Here $f\left( \eta \right)$ is more for more values of $M_{a}$ for injection case. Also $f_{\eta } \left( \eta \right)$ less for more values of $M_{a}$ for $V_{C} < 0$. Figure 10a,b portrays the $f\left( \eta \right)$ verses $\eta$ and $f_{\eta } \left( \eta \right)$ verses $\eta$ for various values of $V_{C}$ respectively. Here $f\left( \eta \right)$ is more for more values of $V_{C}$. Also $f_{\eta } \left( \eta \right)$ less for more values of $V_{C}$.

**Figure 11:**
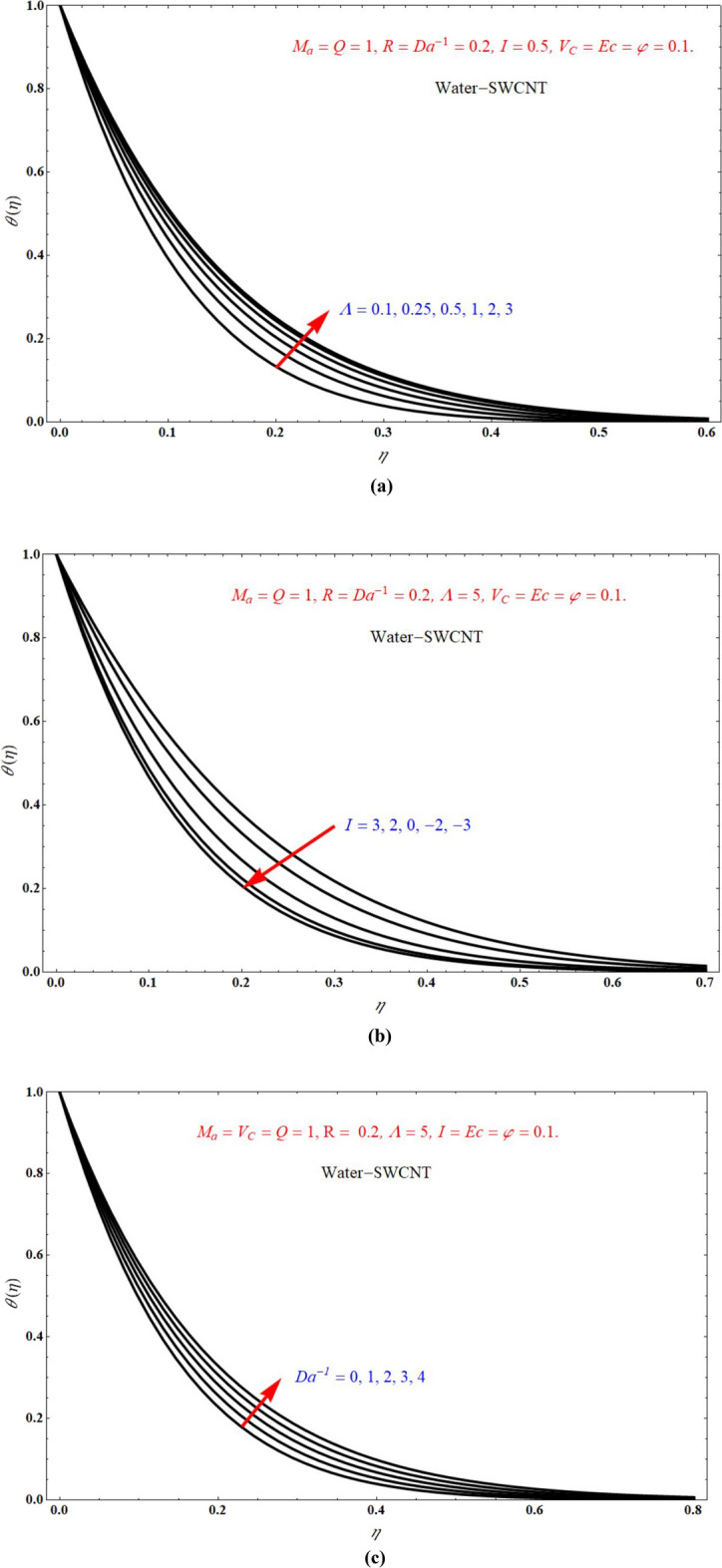
$\theta \left( \eta \right)$ verses $\eta$ for various values of (**a**) $\Lambda$ (**b**) $I$ and (**c**) $Da^{ - 1}$.

**Figure 12:**
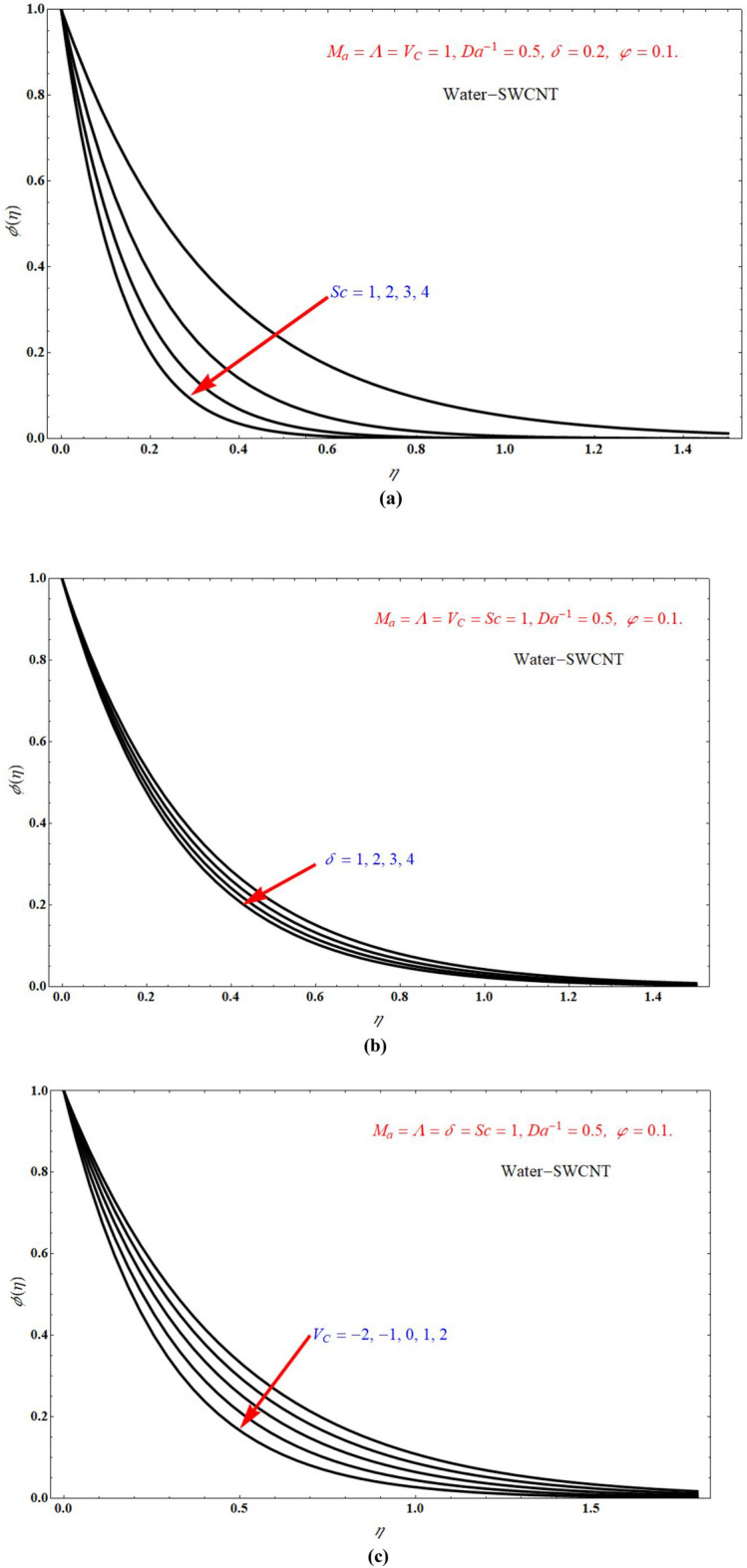
The plots of $\phi \left( \eta \right)$ verses $\eta$ for different choices of (**a**) $Sc$ (**b**) $\delta$ and (**c**) $V_{C}$.

The effect of $\theta \left( \eta \right)$ on $\eta$ for various values of $\Lambda$, $I$, and $Da^{ - 1}$ is respectively represented at Fig. 11a–c. Here, $\theta \left( \eta \right)$ more for more values of $\Lambda$ and $Da^{ - 1}$, but $\theta \left( \eta \right)$ less for more values of $I$. And also we observe that after certain values of $\Lambda$ lines are merging each other. The effect of $\phi \left( \eta \right)$ on $\eta$ for different choices of $Sc$, $\delta$, and $V_{C}$ is respectively represented at Fig. 12a–c. from these graphs it is cleared that $\phi \left( \eta \right)$ decreases with increasing the values of $Sc$, $\delta$, and $V_{C}$. The inclusion of porous media, heat source/sink parameter, thermal radiation and mass transpiration greatly useful in many fields, porous media prevents heat loss/gain and also accelerates the heat source/sink. Heat source/sink results in thinning of the thermal boundary, Marangoni convection results in more induced flows.

## Conclusion

The investigation of results from the 2-D Casson fluid with mass transpiration, thermal radiation and chemically reaction parameter. The ODEs of equations are yielded when we mapped PDEs equation with similarity variables. These ODE equations are solved exactly then the momentum equation is solved to get solution domain, this domain is used in energy and concentration equation to get the temperature profile and concentration profile. The outlook of the present work explains the importance of porous media, thermal radiation, Marangoni convection, thermal radiation and heat source/sink parameter in the physically modelling of the flow. The outcomes we discovered using the graphical scenario are as follows.

1. Effect of the physical solution is directly affected by the $V_{C}$, $Da^{ - 1}$ and $Q$.
2. $\Lambda \,\,{\text{and}}\,\,V_{C}$ directly affected the surface velocity and $Ma$.
3. $f\left( \eta \right)\,\,{\text{and}}\,\,f_{\eta } \left( \eta \right)$ decreases for the instance of suction, injection and no permeability case for rising the values of $\Lambda$ for both suction, injection and no permeability cases.
4. $f\left( \eta \right)$ and $f_{\eta } \left( \eta \right)$ more if we rising the values of $M_{a}$ for suction case.
5. $f\left( \eta \right)$ more for more values of $M_{a}$ for suction case. But $f_{\eta } \left( \eta \right)$ less for more values of $M_{a}$ for injection case. And $f\left( \eta \right)$ more for more values of $V_{C}$ but $f_{\eta } \left( \eta \right)$ less for more values of $V_{C}$.
6. $\theta \left( \eta \right)$ more for more values of $\Lambda$ and $Da^{ - 1}$, but $\theta \left( \eta \right)$ less for more values of $I$.
7. $\phi \left( \eta \right)$ less for more values of $Sc$, $\delta$, and $V_{C}$.
8. Presence of porous media, prevents heat loss/gain and also accelerates the heat source/sink. Chemical reaction term thinning the thermal boundary, Marangoni convection results in more induced flows.
9. The future perspectives of the present work motivate to explain the physically flow problem on the basis of chemically radiative thermosolutal Marangoni convective fluid also helps to conduct flow problems with porous media.
10. Following conditions explain the comparison pf present work with previous works.
  - If $Q = \phi = Ec = 0$(In Eqs. 16 and 17)$\Rightarrow$ Mahabaleshwar et al.^33^.
  - If $Q = \phi = Ec = R = 0$ (In Eqs. 16 and 17) $\Rightarrow$ Mudhaf and Chamkha^8^.
  - If $Q = \phi = Ec = Da^{ - 1} = 0$ (In Eqs. 16 and 17)$\Rightarrow$ Magyari and Chamkha ^14^,

## Data Availability

The datasets used and/or analysed during the current study available from the corresponding author on reasonable request.

## Abbreviations

B. Cs: Boundary conditions
MHD: Magneto hydrodynamics
ODE: Ordinary differential equation
PDE: Partial differential equation

## List of symbols

$B_{0}$: Strength of uniform magnetic field $\left( {{\text{Tesla}}} \right)$
*C*: Concentration of the fluid $\left( {{\text{mol}}\,{\text{m}}^{ - 3} } \right)$
$C_{\infty }$: Concentration of the fluid at infinity $\left( {{\text{mol}}\,{\text{m}}^{ - 3} } \right)$
$C_{w}$: Concentration at wall $\left( {{\text{mol}}\,{\text{m}}^{ - 3} } \right)$
$C_{P}$: Specific heat at constant pressure $\left( {{\text{J}}\,{\text{Kg}}^{ - 1} \,{\text{K}}^{ - 1} } \right)$
*D*: Mass diffusivity $$\left( {{\text{m}}^{2} \,{\text{S}}^{ - 1} } \right)$$
$Da^{ - 1}$: Inverse Darcy number
$G$: Chemical reaction parameter
$I$: Heat source/sink parameter
$K$: The permeability of porous medium $\left( {{\text{m}}^{2} } \right)$
$$L$$: Characteristic length
$$Q$$: Chandrasekhar’s number
$Q_{0}$: Heat source/sink coefficient
$q_{r}$: Radiative heat flux
$T_{\infty }$: Free stream temperature $\left( {\text{K}} \right)$
*T*: Temperature of the fluid $\left( {\text{K}} \right)$
$\left( {u,v} \right)$: Components of velocities $\left( {{\text{ms}}^{ - 1} } \right)$
$V_{0}$: Mass transfer velocity $\left( {{\text{ms}}^{ - 1} } \right)$

## Greek symbols

$\alpha_{r}$: Mean absorption coefficient
$$\beta$$: Solution domain
$$\phi$$: Concentration profile
*μ*: Dynamic viscosity $$\left( {{\text{m}}^{2} \,{\text{s}}^{ - 1} } \right)$$
$$\theta$$: Temperature profile
$$\kappa$$: Thermal conductivity $$\left( {{\text{W}}/{\text{mK}}} \right)$$
$$\sigma$$: Electrical conductivity $$\left( {{\text{Sm}}^{ - 1} } \right)$$
$\sigma_{0}$: Equilibrium surface tension
$\sigma^{*}$: Stefan-Boltzmann constant $\left( {{\text{Wm}}^{ - 2} \,{\text{K}}^{ - 4} } \right)$
$\rho$: Density of the fluid $\left( {{\text{kg}}\,{\text{m}}^{ - 3} } \right)$

## Subscripts

$\infty$: Free stream condition
$\eta$: Differentiation with respect to $\eta$
